# Analytical Evaluation of the Autobio 25-OH Vitamin D Assay: Comparison with Liquid Chromatography–Tandem Mass Spectrometry and Clinical Implications

**DOI:** 10.3390/metabo15120802

**Published:** 2025-12-18

**Authors:** Flaminia Tomassetti, Eleonora Nicolai, Martina Pelagalli, Federico Cortese, Alfredo Giovannelli, Sabrina Ballerini, Alessia Mozzi, Anastasia De Luca, Massimo Pieri, Sergio Bernardini

**Affiliations:** 1 Department of Experimental Medicine, University of Rome Tor Vergata, Via Montpellier 1, 00133 Rome, Italy; flaminia.tomassetti@students.uniroma2.eu (F.T.); alfredo.giovannelli@gmail.com (A.G.); bernards@uniroma2.it (S.B.); 2 Department of Laboratory Medicine, Tor Vergata University Hospital, Viale Oxford 81, 00133 Rome, Italy; pelagallimartina90@gmail.com (M.P.); sabrina.ballerini@ptvonline.it (S.B.); alessiafrancesca.mozzi@ptvonline.it (A.M.); anastasia.deluca@uniroma2.it (A.D.L.); 3 Department of Biomedicine and Prevention, University of Rome Tor Vergata, 00133 Rome, Italy; 4 Departmental Faculty of Medicine, UniCamillus-Saint Camillus International University of Health and Medical Sciences, Via di Sant’Alessandro, 8, 00131 Rome, Italy; eleonora.nicolai@unicamillus.org; 5 Department of Electronic Engineering, University of Rome Tor Vergata, 00133 Rome, Italy; federico.cortese@alumni.uniroma2.eu; 6 Department of Industrial Engineering, University of Rome Tor Vergata, 00133 Rome, Italy; 7 Department of Biology, University of Rome Tor Vergata, 00133 Rome, Italy

**Keywords:** vitamin D, hypovitaminosis, method validation

## Abstract

Background: 25-Hydroxy vitamin D [25(OH)D] is the circulating form of vitamin D. Its deficiency is a major global health concern, affecting over one billion people. Beyond its role in bone health, low vitamin D levels have been implicated in a wide range of chronic and inflammatory diseases, including diabetes, chronic kidney disease, and cancer. While immunoassays are widely used in routine testing, liquid chromatography–tandem mass spectrometry (LC-MS/MS) remains the reference method for its superior accuracy. This study aimed to evaluate the analytical performance of the Autobio 25(OH)D chemiluminescence assay (Autobio Diagnostics, Zhengzhou, China) compared with LC-MS/MS (Chromsystems Instruments & Chemicals GmbH, Gräfelfing, Germany) and the Siemens chemiluminescent microparticle immunoassay (Siemens HealthCare, Erlangen, Germany). Additionally, the influence of age and sex on 25(OH)D concentrations was examined to explore potential demographic and pathophysiological variations. Methods: 200 residual serum samples were analyzed to compare all three methods. Precision and linearity were verified. Statistical analysis was performed. Results: The Autobio assay showed good correlation with LC-MS/MS (R^2^ = 0.953; *p* < 0.001), with acceptable bias and precision (CV < 10%) and confirmed linearity. Age- and sex-related differences were observed, indicating demographic influences on vitamin D status. Conclusions: Accurate and accessible laboratory testing for 25(OH)D is therefore essential for both disease prevention and clinical management. The Autobio 25(OH)D assay demonstrated strong correlation with LC-MS/MS and high analytical reliability. Its good performance makes it a valuable tool for routine assessment of 25(OH)D and for supporting the early detection or monitoring of hypovitaminosis D in clinical practice.

## 1. Introduction

Vitamin D is a fat-soluble secosteroid [1] that functions as a prohormone and plays a fundamental role in calcium and phosphate homeostasis, bone mineralization, and overall skeletal integrity [2]. It exists primarily as ergocalciferol (vitamin D_2_), from plant sources, and cholecalciferol (vitamin D_3_), synthesized in the skin upon exposure to ultraviolet B (UVB) radiation or obtained through diet and supplements.

Beyond its classical role in bone health, vitamin D is gaining recognition for its involvement in various non-skeletal functions. It modulates immune responses and plays a role in cardiovascular protection and the prevention of chronic diseases, including autoimmune disorders, metabolic syndromes, and certain malignancies [2]. These pleiotropic effects are mediated in part through the vitamin D receptor (VDR), a member of the nuclear hormone receptor superfamily [3]. Activated by 1,25-dihydroxyvitamin D [1,25(OH)_2_D], VDR regulates gene transcription and can modulate pathways such as CREB (cAMP-response element-binding protein), thereby influencing inflammatory and immune responses [4].

The principal circulating metabolite, 25-hydroxyvitamin D [25(OH)D], is synthesized in the liver and serves as a reliable biomarker for assessing vitamin D status. Despite the growing awareness, vitamin D deficiency and insufficiency remain global health concerns affecting over one billion people worldwide, with prevalence rates ranging from 50% to 80%, particularly among elderly individuals, pregnant women, and individuals with limited sunlight exposure [5].

Epidemiological studies highlight the association between suboptimal vitamin D levels and increased susceptibility to immune-mediated and infectious diseases. Vitamin D deficiency is typically defined as a serum 25(OH)D concentration below 20 ng/mL (severe if levels < 10 ng/mL), while levels between 20 and 30 ng/mL are considered insufficient, often without evident clinical symptoms [6,7,8]. These suboptimal levels (20 and 30 ng/mL) are linked to acute and chronic conditions, including preeclampsia, autoimmune diseases, cardiovascular disorders, type 2 diabetes, tumors, malabsorption syndromes, systemic inflammation, and the use of medications such as anticonvulsants, rifampicin, or antiretroviral therapies [9,10,11,12,13,14,15]. Vegetarians, individuals with limited sun exposure, and those with photosensitive skin disorders are at an increased risk [16]. In response, the WHO and FAO recommended a multifaceted approach to improve vitamin D status globally, including dietary diversification, food fortification, and targeted supplementation.

Various analytical methods exist for measuring serum 25(OH)D. Competitive binding immunoassays, including chemiluminescent microparticle immunoassays (CMIA), chemiluminescence immunoassays (CLIA), and radioimmunoassays (RIA), are commonly employed due to their speed, automation, and cost-effectiveness. They are limited by cross-reactivity with vitamin D metabolites and interassay variability. Conversely, liquid chromatography-tandem mass spectrometry (LC-MS/MS) is the gold standard because of its high specificity and capacity to distinguish between 25(OH)D_2_ and 25(OH)D_3_ [17,18]. However, its high cost, complexity, and limited accessibility restrict its use in routine laboratory settings.

Given these challenges, there is a pressing need for accurate, reproducible, and scalable assays that can reliably assess vitamin D status in diverse patient populations. This study presents the clinical validation of a newly developed CLIA test by Autobio (Autobio Diagnostics, Zhengzhou, China). We compared its analytical performance with that of LC-MS/MS (ChromeSystem, Munich, Germany) and a widely used commercial CMIA assay (Siemens, Munich, Germany), focusing on method agreement, linearity, and precision. We also examined the influence of demographic factors, such as age and sex, on serum 25(OH)D levels.to assess whether the Autobio assay is a reliable and practical alternative for routine use in clinical diagnostics.

## 2. Materials and Methods

### 2.1. Samples Collection

The study was conducted using 200 residual routine serum samples collected from the daily routine. The samples were drawn into an SST (Serum Separator Tube) with Gel-Tube, and after clotting (usually 15–30 min), were centrifuged at 2000× *g* for 10 min in a refrigerated centrifuge. The resulting supernatant is designated as serum. The sera were anonymized and selected to represent a broad demographic stratified by age group and gender. The samples were aliquoted and stored at −80 °C until the work session. Inclusion criteria were based on sample availability and completeness of demographic data. Exclusion criteria were heat- or light-inactivated samples, pooled samples, hemolyzed samples (<1 g/L of hemoglobin), and samples not stored at refrigerated temperatures (2–8 °C).

Ethical consent was obtained in accordance with the Helsinki Declaration and approved by the Ethics Committee of University of Rome Tor Vergata (Registration Number: 141/20).

### 2.2. Chemiluminescence Assay, CLIA (Autobio)

The Autobio 25(OH)D assay is a two-step sandwich chemiluminescence immunoassay (CLIA) performed on the AutoLumo A1860 platform (Autobio Diagnostics, Zhengzhou, China). The assay, based on a two-step sandwich method, quantifies total 25(OH)D (including D_2_ and D_3_). As first step, 25(OH)D antibody-coated microparticles and dissociation solution are added to the sample. During incubation, the 25(OH)D antigen in the sample binds to the antibody-coated microparticles. After washing, the enzyme conjugate is added to the reaction mixture. During incubation, a complex forms between the microparticles, the 25(OH)D antigen in the sample, and the antibodies bound to the enzymes by immunological reactions. The chemiluminescent substrate is added, and the complex catalyzes the substrate, causing a chemiluminescent reaction. The resulting chemiluminescence is measured as the relative light units (RLU). The RLU is proportional to the amount of 25(OH)D in the samples. Detailed information of this assay is summarized in Table 1.

Quality control materials at two concentration levels were performed before every analytical session.

### 2.3. Chemiluminescent Microparticle Immunoassay, CMIA (Siemens)

The Atellica IM 25(OH)D assay (Siemens HealthCare, Erlangen, Germany) is a competitive Chemiluminescent Microparticle Immunoassay (CMIA) that uses an anti-fluorescein mouse monoclonal antibody covalently bound to paramagnetic particles (PMP), an anti-25(OH)D mouse monoclonal antibody labeled with acridinium ester (AE), and a vitamin D analog labeled with fluorescein. An inverse relationship exists between the amount of vitamin D present in the patient sample and the amount of RLU detected by the system. Detailed information of this assay is summarized in Table 1.

### 2.4. Liquid Chromatography–Tandem Mass Spectrometry (Chromsystem)

Liquid Chromatography–Tandem Mass Spectrometry (LC-MS/MS) was used as the reference method. Serum samples were analyzed for the total 25(OH)D (the sum of 25(OH)D_2_ and 25(OH)D_3_). The reagent kit was provided by Chromsystems Instruments & Chemicals GmbH (Munich, Germany): LC-MS/MS Reagent Kit MassChrom^®^ 25(OH)D3/D2 in serum/plasma, Order No. 62000/1000. The kit included the reagents and materials for sample preparation, the analytical column, trap column, and the lyophilized samples for calibration curves and controls. Samples were prepared according to the kit’s instruction manual and then subjected to LC-MS/MS analysis. This was performed using a Shimadzu Nexera X3 (Shimadzu, Kyoto, Japan) high-performance liquid chromatography (HPLC) system coupled to the Sciex Triple Quad ^®^ 6500 + MS/MS system (Sciex, Framingham, MA, USA). All analyses were carried out in positive mode using atmospheric pressure chemical ionization (APCI). For quantitative analyses, multiple reaction monitoring (MRM) was applied. Of the two options available for chromatographic analysis, a binary gradient was used. Detailed information about the transitions employed and the chromatographic setup is freely available on the Chromsystems website.

Table 1 summarizes the main technical features and operating principles of the three methods.

### 2.5. Comparison Analysis

Passing Bablok correlation analysis was used to compare the three methods, employing the nonparametric measure of rank correlation (statistical dependence between the rankings of two variables) and the Spearman rank correlation coefficient (R^2^). Bland–Altman test was performed as an alternative analysis to determine the agreement between the different assays concerning the gold standard (LC-MS/MS) and to investigate any possible relationship of the discrepancies between the measurements and the true value.

### 2.6. Precision Study

The precision study was conducted according to the CLSI EP15-A3 guidelines [19], replicating each control level 10 times. Coefficients of variation (CVs) were determined as the standard deviation divided by the mean value, expressed as a percentage. The CVs calculated were compared to those provided by the manufacturer.

### 2.7. Linearity Evaluation

To confirm linearity, samples at eight different concentrations within the expected range (declared concentration range 3.0–150.0 ng/mL) were employed. High-concentration samples were prepared by combining samples at a concentration close to the linearity threshold and subsequently diluting them with the manufacturer’s diluent. Each concentration was tested twice. The mean and standard deviation were calculated, and a linear regression analysis was performed to fit the mean values and dilution ratios using the least squares method. Lastly, the correlation coefficient r was computed within the linear range.

### 2.8. Statistical Analysis

For normal data distribution, parametric tests were used, such as analysis of variance (ANOVA) with a Bonferroni post hoc test for more than two variables. For the non-normal data distribution non-parametric test, the Kruskal–Wallis test (variables with more than two categories) was used to verify differences between groups.

All statistical analyses were performed using MedCalc (version 18.2.1, MedCalc Software, Ostend, Belgium) and R software (version 4.5.1, Posit, Boston, MA, USA), with significance set at *p* < 0.05.

## 3. Results

### 3.1. Comparison Analysis

**Figure 1:**
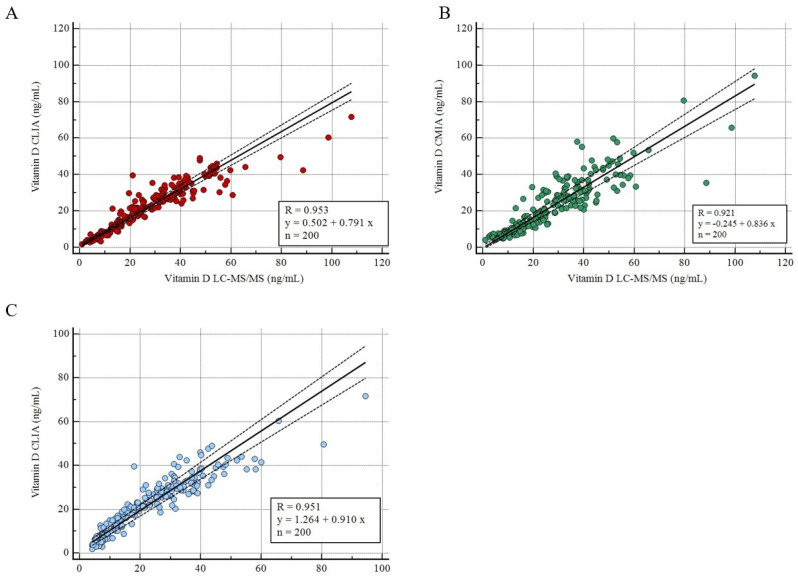
Correlation analysis. (**A**) Passing Bablok between LC-MS/MS and CLIA methods. (**B**) Passing Bablok between LC-MS/MS and CMIA methods. (**C**) Passing Bablok between CMIA and CLIA methods.

The results of serum 25(OH)D levels measured using the CLIA, CMIA, and LC-MS/MS methods are shown in Figure 1. The Passing Bablok analysis revealed a strong correlation among all methods. The regression equation between LC-MS/MS and CLIA, in Figure 1A, revealed an R of 0.953 (*p* < 0.001), with an intercept A of 0.502 (95% Confidence Interval, CI: −0.1688 to 1.252) and a slope B of 0.791 (95% CI: 0.755 to 0.825). Likewise, the regression equation between LC-MS/MS and CMIA, in Figure 1B, discovered a similar R of 0.921 (*p* < 0.001), with an intercept A of −0.245 (95% CI: −1.236 to 0.987) and a slope B of 0.836 (95% CI: 0.770 to 0.901). Lastly, the regression equation between LC-MS/MS and CLIA, in Figure 1C, assessed an R of 0.951 (*p* < 0.001), with an intercept A of 1.264 (95% CI: 0.038 to 2.443) and a slope B of 0.910 (95% CI: 0.846 to 0.977).

**Figure 2:**
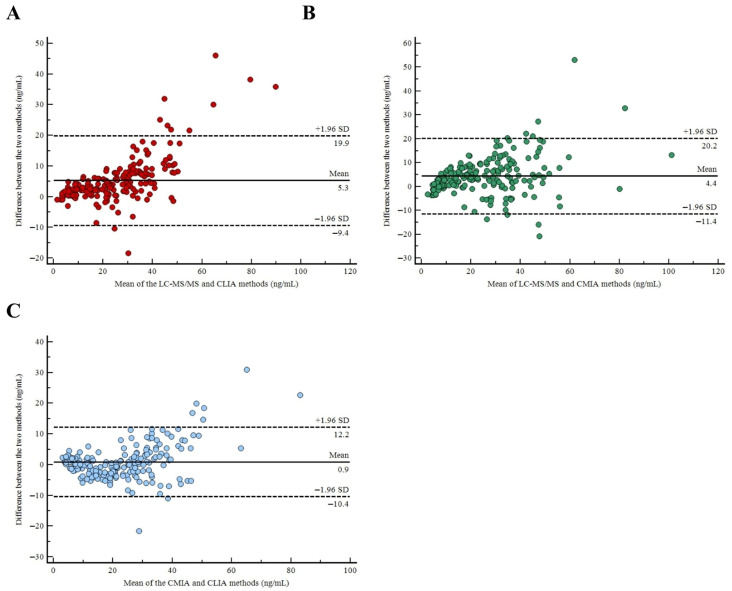
Methods agreement. (**A**) Bland–Altman between LC-MS/MS and CLIA methods. (**B**) Bland–Altman between LC-MS/MS and CMIA methods. (**C**) Bland–Altman between CMIA and CLIA methods.

Moreover, to assess method agreement, the Bland–Altman analysis was conducted. The mean bias between the results of 25(OH)D measured by the LC-MS/MS and CLIA method was 5.3 (minimum −9.4; maximum 19.9), indicating that the LC-MS/MS method performs higher results than the CLIA (Figure 2A). Instead, the mean bias between the results of 25(OH)D measured by the LC-MS/MS and CMIA method was 4.4 (minimum −11.4; maximum 20.2), indicating that the differences between methods exhibit homoscedasticity, i.e., the spread of the data around the regression line is approximately the same (Figure 2B). To conclude, the mean bias between the results of 25(OH)D measured by the CMIA and CLIA method was 0.9 (minimum −10.4; maximum 12.2), indicating a quite small average discrepancy, suggesting acceptable agreement (Figure 2C).

### 3.2. Precision Study

The precision of the analytical method was evaluated by analyzing ten replicate measurements of both low- and high-concentration quality controls (QC) on the same day under identical conditions. The low QC yielded to a mean value of 14 with a SD of ±0.52, corresponding to a CV of 3.82%. The high QC produced a mean of 76 with a SD of ±1.82, resulting in a CV of 2.39%. Both CVs were well below the CV declared by the producer (<15%), indicating excellent method precision and reproducibility across the tested concentration range.

### 3.3. Linearity Evaluation

**Figure 3:**
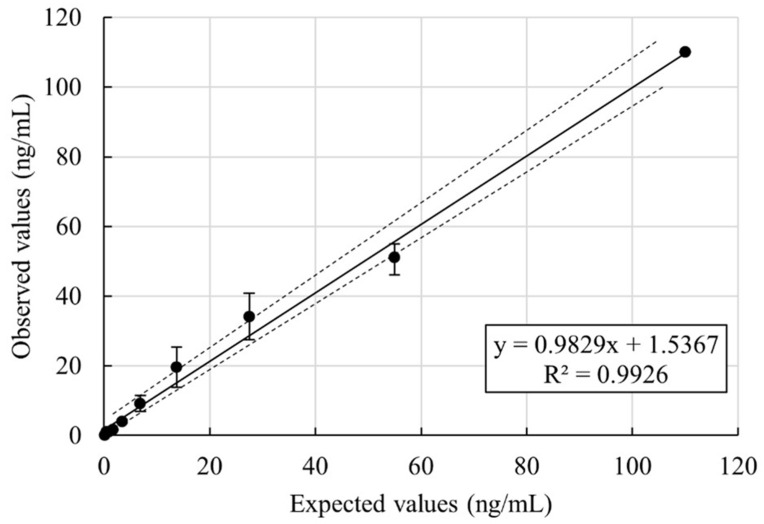
Linearity study. [Continuous line: regression line; dashed line: 95% Interval confidence; error bars: Standard Deviation, SD].

The linearity assessment of the Autobio 25(OH)D is presented in Figure 3. Serial dilutions were prepared from high-concentration serum pools, to cover the declared linearity range (3.0–150.0 ng/mL) with the manufacturer’s diluent. All tested dilution points demonstrated linearity, with no significant deviation from expected values. The resulting regression equation was built on the mean of the two sets of serial dilution, y = 0.98 + 1.53x, with an R^2^ = 0.99, indicating a strong linear relationship across the tested range.

### 3.4. Population

**Figure 4:**
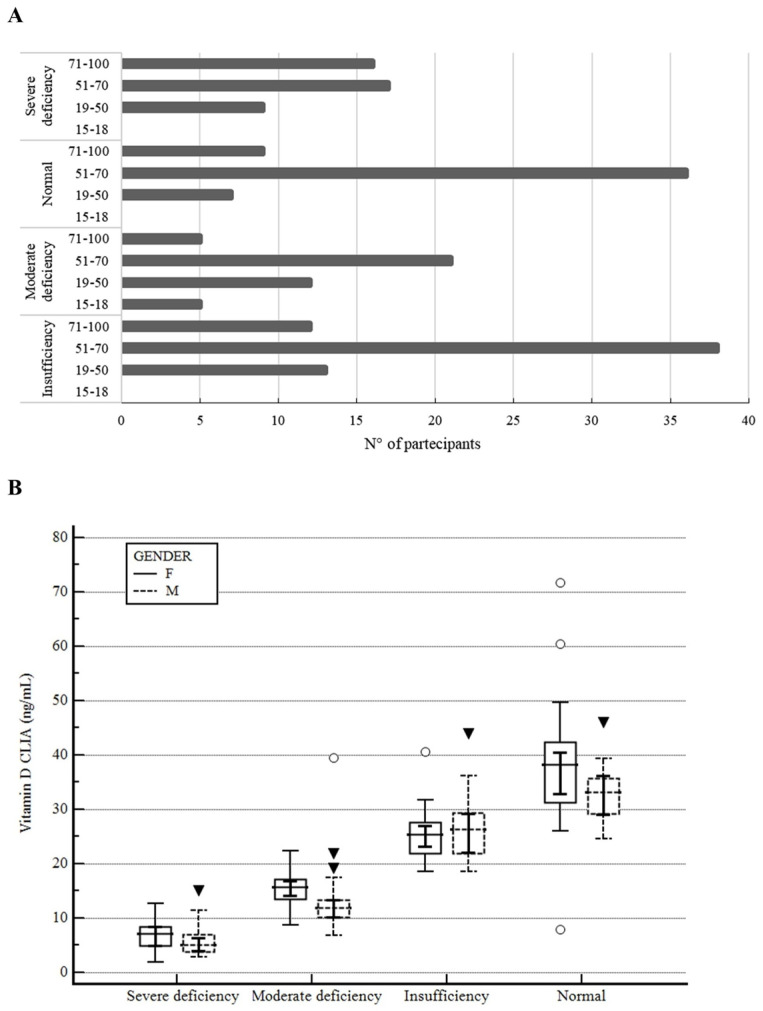
(**A**) Age distribution of Vitamin D levels across groups. (**B**) Gender-based distribution of Vitamin D levels across groups. Although there are differences between males and females in the four groups, no statistical difference was observed (*p*: non-significant). [Continuous line: females; dashed line: males; upside-down triangle: outliers; horizontal line: mean; error bars: Standard Deviation, SD].

The analysis included 200 individuals stratified by age and sex, with biomarker concentrations (evaluated by CLIA method, ng/mL) showing variable patterns across age groups. Figure 4A illustrates the distribution of serum Vitamin D concentrations (CLIA, ng/L) across four age categories (15–18, 19–50, 51–70, and 71–100 years), stratified by groups, and a clear age-related pattern emerged, with the highest prevalence of Vitamin D insufficiency observed in the elderly population. Notably, severe and moderate deficiencies were more frequent in younger and middle-aged adults (19–50 years), suggesting early onset of suboptimal Vitamin D status. Instead, the 71–100 years group showed a more balanced distribution, though deficiency remains prevalent, possibly reflecting increased supplementation or clinical monitoring in older populations.

In the 15–18-year age group, the female participants (N = 5) had a mean concentration of 17.17 ± 4.01 ng/mL, with values ranging from 11.97 to 22.31 ng/mL. Just one male participant was observed (Vitamin D 11.97 ng/mL).

In the 19–50-year group, comprising 28 females and 13 males, the mean concentration among females was 24.12 ± 14.85 ng/mL, while males showed a lower mean of 15.91 ± 8.86 ng/mL. The total group mean was 27.52 ± 13.68 ng/mL, with concentrations ranging from 3.42 to 71.79 ng/mL.

Among individuals aged 51–70 years, biomarker levels remained elevated. Females (N = 68) had a mean value of 26.52 ± 11.68 ng/mL, whereas males (N = 44) demonstrated a slightly lower mean of 20.31 ± 11.96 ng/mL. The combined group mean was 24.08 ± 12.12 ng/mL, with individual values spanning from 1.89 to 60.46 ng/mL.

In the oldest group (71–100 years), biomarker concentrations showed a slight decline compared to the previous age group. Females (N = 16) presented a mean level of 21.83 ± 13.94 ng/mL, while males (N = 26) showed a mean of 16.33 ± 13.18 ng/mL. The overall group mean was 18.42 ± 13.58 ng/mL, with values ranging from 2.91 to 49.03 ng/mL.

Furthermore, the population was divided into four categories based on Vitamin D value, as suggested by the guidelines [7,8]: severe deficiency (<10 ng/mL), moderate deficiency (10–20 ng/mL), insufficiency (20–30 ng/mL), and normal status (30–50 ng/mL).

Figure 4B displays the gender differences between the groups. Across all categories, females consistently exhibited higher mean Vitamin D levels compared to males, suggesting a sex-related disparity in Vitamin D status; however, no statistically significant difference was observed. In severe deficiency, female values (*n* = 15) were slightly higher than male values (*n* = 27) with a mean of 6.83 ± 1.61 vs. 5.77 ± 1.19. The moderate category presented a wider spread for both sexes, though females (*n* = 25) maintained higher central values with a mean of 15.36 ± 1.24 than males (*n* = 18), mean of 13.93 ± 1.46. Instead, the two genders in the insufficiency group showed a similar mean with a wider spread in the values: female (*n* = 37) mean of 25.61 ± 1.02; male (*n* = 26) 26.78 ± 1.22. Lastly, in the normal group, Vitamin D levels were substantially elevated in both sexes, yet females (*n* = 39) still demonstrated a higher mean of 37.63 ± 0.99 than males (*n* = 13), a mean of 33.41 ± 1.73, reinforcing the observed trend.

## 4. Discussion

Vitamin D is vital for health at all life stages [20], aiding in calcium and phosphorus balance, bone mineralization, and influencing the immune, endocrine, and cardiovascular systems. Growing evidence from several studies suggests that sufficient vitamin D is crucial for optimal health [20,21]. Low vitamin D levels increase the risk of osteoporosis, fractures, autoimmune and infectious diseases, and cardiometabolic conditions [21,22]. Nonetheless, nowadays, vitamin D deficiency is common globally due to contemporary lifestyle factors, including reduced sunlight exposure, increased sedentary behavior, and dietary insufficiency, which frequently result in serum 25(OH)D concentrations that are inadequate to meet physiological demands. This discrepancy underscores the importance of continuous evaluation and harmonization of assay performance.

The evaluation of vitamin D status is primarily performed by measuring circulating 25(OH)D, which is the most reliable biomarker of vitamin D exposure. While LC-MS/MS is widely regarded as the reference method owing to its analytical specificity, its application in routine clinical practice is constrained by technical complexity, substantial capital investment, and the requirement for highly trained personnel. For these reasons, automated immunoassay platforms remain the main approach in clinical laboratories, where they provide high-throughput, cost-efficient, and rapid testing. For this, the present study evaluated a new CLIA system by Autobio (Autobio Diagnostics, Zhengzhou, China).

Specifically, correlation analysis demonstrated strong agreement between both the CLIA and LC-MS/MS (R = 0.953), and the CLIA and the CMIA (R = 0.951), indicating robust analytical comparability. Importantly, no significant systematic bias was observed, suggesting that the Autobio platform may provide clinically interchangeable results with the reference method in most cases.

Precision was further assessed by repeated analysis of low- and high-level quality control samples, with CVs consistently below 15%. These results align with internationally accepted quality specifications and previous studies [17,23] and affirm the assay’s repeatability and suitability for routine clinical application. Linearity testing confirmed accurate performance across the clinically relevant measurement range, thereby supporting its reliability for identifying both deficient and sufficient serum 25(OH)D levels.

Subgroup analyses provided additional insights into population trends. Age and sex stratification revealed lower mean 25(OH)D levels among older adults and male participants, consistent with previous reports. These demographic patterns highlight the necessity of contextualizing laboratory results with patient-specific factors, particularly when applying generalized reference intervals. The age-related decline in cutaneous vitamin D synthesis, combined with reduced dietary intake and diminished outdoor activity, likely contributes to the increased prevalence of deficiency in elderly populations. Sex-based differences in vitamin D metabolism contrast with existing literature. Women tend to be more proactive about their health [24], which may partially explain the relatively higher serum levels observed in females. Indeed, women are often more knowledgeable about the menopausal transition and osteoporosis risk and more likely to engage in health promotion interventions. Such screening programs, along with active preventive measures for bone health—including vitamin D supplementation, calcium intake monitoring, and lifestyle interventions—may further contribute to the more favorable vitamin D status frequently observed in female cohorts.

While LC-MS/MS remains the more accurate method, it can discriminate between the two Vitamin D isoforms; the practical advantages of high-throughput immunoassays, such as CLIA, should be considered. The Autobio assay demonstrated performance characteristics, and the results obtained highlighted it as a viable alternative in routine clinical laboratories.

It is important to acknowledge some limitations. The seasonal fluctuation in 25(OH)D levels, a well-documented phenomenon resulting from changes in sunlight exposure, was not systematically addressed [25,26,27,28,29].

## 5. Conclusions

In conclusion, our study emphasizes the prevalence of vitamin D deficiency in our population and the related misinformation. There is growing evidence supporting age-specific screening protocols and supplementation guidelines to alleviate the long-term health impacts of persistent vitamin D deficiency. These strategies may include dietary fortification, structured supplementation protocols, and increased public knowledge. Moreover, this work demonstrated the high performance and good reliability in clinical settings of the new Autobio Vitamin D test. These findings reinforce the need for continuous evaluation of automated immunoassays and contribute to the efforts to optimize vitamin D testing in medical practice.

## Figures and Tables

**Table 1:** Key characteristics of the three methods: CLIA, CMIA and LC-MS/MS. [LOB: Limit of Blank; LOD: Limit of Detection; LOQ: Limit of Quantification; CV: Coefficient of Variation].

	CLIA	CMIA	LC-MS/MS
Sample volume	50 µL	20 µL	100 µL
Matrices	Serum and plasma (EDTA, lithium heparin, and sodium heparin)	Serum and plasma (EDTA, lithium heparin, and sodium heparin)	Serum and plasma (EDTA, lithium heparin, and sodium heparin)
LOB	1.0 ng/mL	1.70 ng/mL	25(OH) D3: 1.4 µg/L 25(OH) D2: 1.1 µg/L
LOD	2.0 ng/mL	3.20 ng/mL	25(OH) D3: 1.4 µg/L 25(OH) D2: 1.1 µg/L
LOQ	3.0 ng/mL	4.20 ng/m	25(OH) D3: 1.4 µg/L 25(OH) D2: 1.1 µg/L
Measuring interval	3.0–150.0 ng/mL	4.20–150.00 ng/mL	25(OH) D3: 1.4–250 µg/L 25(OH) D2: 1.1–250 µg/L
Precision	<3.2% CV	≤12% CV	25(OH) D3: 3.3% CV 25(OH) D2: 4.6% CV

## Data Availability

Data are available under reasonable request.
